# Building resilience to El Niño‐related drought: experiences in early warning and early action from Nicaragua and Ethiopia

**DOI:** 10.1111/disa.12340

**Published:** 2019-04-04

**Authors:** Richard Ewbank, Carlos Perez, Hilary Cornish, Mulugeta Worku, Solomon Woldetsadik

**Affiliations:** ^1^ Climate Advisor at Christian Aid United Kingdom; ^2^ Senior Programme Officer for Resilience, Christian Aid Central America; ^3^ Research, Evidence and Learning Advisor at Christian Aid United Kingdom; ^4^ Value Chain Development Project Manager at Oxfam Ethiopia; ^5^ Senior Programme Officer, BRACED (Building Resilience and Adaptation to Climate Extremes and Disasters), Christian Aid Ethiopia

**Keywords:** drought, early action, early warning, El Niño, resilience

## Abstract

Forecast‐based drought early warning/early action has been hampered by both inadequate decision‐making frameworks and a lack of appropriate funding mechanisms. Rural communities in Nicaragua and Ethiopia that have participated in resilience‐building interventions of varying durations demonstrate the value of community‐based actions informed by early warning, forecasts and drought management advice, both before and during the agricultural season. While drought affected all crops negatively, participants were better able to mitigate impacts, were more organised in accessing relief and recovered more effectively. These results are consistent with other research on the cost/benefit of anticipatory actions, use of climate services and appropriate drought management advice. They also confirm the importance of embedding short‐term early action in long‐term resilience‐building. Despite this, formal systems, national and local, remain essentially unimplemented. Systems being developed at global level now need to be operationalised and translated into effective local drought management standard operating procedures for the most vulnerable.

## The effectiveness of resilience and early action

Early warning and early action systems have been widely used to manage fast‐onset climate shocks, such as cyclones, but application to slower‐onset emergencies such as drought has proved more problematic. Despite substantial improvements in El Niño–Southern Oscillation (ENSO) and drought forecasting since the late 1980s, delivering an early action period of three to twelve months depending on seasonality, assessments of responses as recent as that in the 2011 East African drought highlight persistent failure to adopt and implement early warning and early action (Bailey, 2012). Obstacles include decision frameworks that are ‘hard wired for delay', with incentives geared towards avoiding the downsides of a false alarm rather than the benefits of early, risk‐mitigating, action. Even if this were not the case, there is a lack of pooled funds and financing mechanisms that could disburse sufficient resources based on agreed thresholds. This contrasts with other important development priorities, covered by initiatives such as the Global Fund for HIV, Tuberculosis and Malaria, the Global Environment Facility and the Central Emergency Response Fund.^2^

Increasing evidence has demonstrated the short‐sightedness of this. For example, a study for the United Nations Children's Fund (UNICEF) and the World Food Programme (WFP) (Meerkat et al., 2017) shows that all preparedness investments have saved cost and/or time, with an average of over $2 saved in humanitarian cost for every $1 spent. Earlier research (Cabot Venton et al., 2012) found that investment in resilience brought substantial returns in terms of need averted and broader developmental outcomes, with benefit to cost ratios of $2.30–$13.20 for each $1 spent. The same study also suggested that concerns over triggering false alarms were unwarranted, with the costs associated with a single late response equating to those of two to six false alarms. For early warning systems specifically (Wethli, 2014), a median benefit to cost ratio of $5:$1 confirms that anticipatory risk management is often overwhelmingly the most cost‐effective option.

Experience with climate services has similarly demonstrated the value of forecast‐based decision‐making. In Zimbabwe, the use of seasonal forecasts and participatory workshops with farmers delivered a 9.4% increase in harvests across two years (Patt et al., 2005). In Mali, yield results for farmers taking management decisions using agro‐meteorological information suggested increases of 37% and 36% for millet and sorghum, respectively (Helmuth et al., 2010). Assessments of the most effective options with respect to extreme weather highlight the importance of long‐term approaches to drought resilience, citing agro‐ecological management practices, soil and water conservation, improved wells and agroforestry as the most effective ways to manage drought risk (Royal Society, 2014).

## Evolution of the 2015–16 El Niño

ENSO is ‘a naturally occurring phenomenon involving fluctuating ocean temperatures in the central and eastern equatorial Pacific, coupled with changes in the atmosphere’ (WMO, 2014). El Niño describes the warm phase of the cycle, with La Niña the cool phase. The cycle generally lasts two to seven years and influences climatology across 100 mainly tropical and sub‐tropical countries.

The origins of the 2015–16 El Niño emerged in April 2014, when precursors were detected in the tropical Pacific. By September, certainty had declined to 60% and, although by November thresholds for a weak El Niño had been exceeded, atmospheric variables remained near average. This situation persisted through to March 2015, with caution over forecasts of El Niño emerging mid‐year owing to the lower skill of ENSO forecasts in the first quarter (before the ‘spring barrier') and its failure to emerge fully in 2014. This situation changed during April as probabilities for El Niño increased to 70%. By September, it was clear that a strong El Niño was underway^3^ (WMO, 2015).

## Drought impacts in Nicaragua and Ethiopia

Central America's drought started in mid‐2014, affecting 500,000 farmers across the region, and by September 460,000 people were estimated to be in moderate to severe food insecurity (WFP, 2015). In early 2015, as El Niño strengthened, the number of people requiring emergency support regionally increased to 2.8 million, with about 346,500 of them in Nicaragua (DFID, 2015). The *primera* rains (May–July) arrived late and, apart from some short‐lived relief in early June, were substantially below average. In the north‐western part of the country, less than 40% of average July rainfall was received, leading to an early *canicula* (dry period, usually from mid‐July to mid‐August) before any crops had matured. This pattern continued into the *postrera* (August–October), with rainfall delayed by 20–30 days (FAO GIEWS, 2015).

Nicaragua's 2014 maize harvest was 21% below the five‐year average, and the figure dropped to 38% below in 2015. Rainfall in Somotillo (western dry corridor) demonstrated the extended nature of the deficit, with depressed rainfall in 2013 and 2014 intensifying to just 44% of the long‐term average in 2015. Unlike in neighbouring countries, the government did not declare an emergency. In September 2015, it began distributing food to 27,000 households and agricultural technology packages to 23,000. Compounding drought was the coffee rust outbreak of 2012–14. About 70% of Nicaragua's coffee is grown by small‐scale farmers, and, with 50% of the growing area affected, production dropped by 20% in 2014. Casual labour demand contracted by 14–22% (FEWS NET et al., 2014). This has particularly affected households in the dry corridor, which devote part of the year to seasonal earning opportunities in coffee areas.

Drought impacts in Ethiopia evolved primarily in the eastern and north‐eastern areas of the country. Total average rainfall across this area averaged 480 mm between March and September 2015, the lowest level for 50 years, leading water availability per capita to drop to below 35% of the average. Both the *belg* (February–May) and the *kiremt* (June–September) seasons delivered severely depressed levels of rainfall. At the beginning of June, the government declared *belg* failure and the resulting assessment identified 4.5 million people in need of food assistance in August. As the *kiremt* season also resulted in severely depressed yield – cereal yields in Dire Dawa (adjacent to the Kombolcha area assessed in this research) were 45% down on the previous year (CSA, 2016) – this rose to 8.2 million by mid‐October before peaking in December at 10.1 million people.

**Figure 1:**
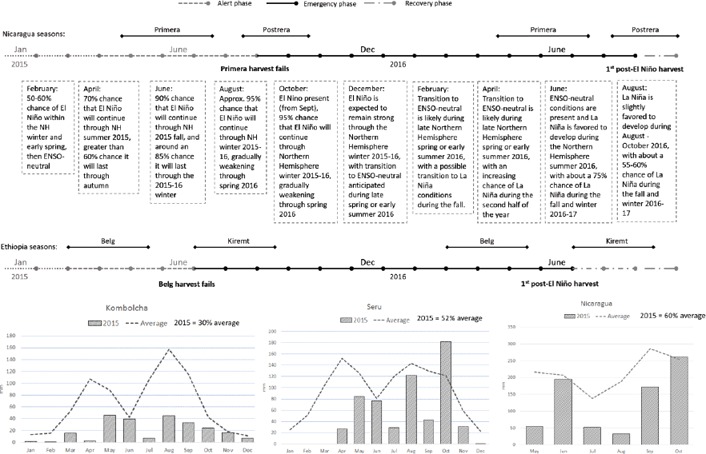
Evolution of El Niño and impact on rainfall^4^ **Source**: authors.

In August 2015, Ethiopia's humanitarian requirements reached $432 million, and this had risen to $596 million by October, with about 43% of it covered. The government allocated $192 million. The extent of severity was illustrated by the number of *woredas* (districts) prioritised for nutrition interventions, which jumped from 96 in July to 143 by September (UN EHCT, 2015). The emergency response involved 43 international non‐governmental organisations (NGOs), 13 national NGOs, 10 United Nations and 3 Red Cross agencies (NDRMC and UN EHCT, 2015) and an estimated $1.4 billion in funding.

As in Nicaragua, farmers have also had to contend with an increase in fungal crop disease, here mainly yellow wheat rust, which typically causes 50–100% crop loss if left untreated. After abating during the drought, this returned in the 2016 recovery seasons, with 45,000 small‐scale farmers affected during the *kiremt* (including Seru, one of the two survey sites). This was the worst outbreak since 2011. By November, 92% of affected farms had received some fungicide treatment (FAO, 2016).

## Background and methodology

The theory of change that guided this assessment was as follows:

*through supporting communities to develop risk‐based plans and implement resilience‐building activities, including increasing access to climate information and forecasts, they are better able to cope with and reduce the impact on their livelihoods of the drought that resulted from the 2015–16 El Niño*.

In both countries, sites were selected according to their drought vulnerability, with two areas chosen – one a traditionally drought‐vulnerable area and one less so – to broaden the representativeness of the research, increase the diversity of responses and, where relevant, enable the comparison of sites. In Nicaragua, Somotillo is located in the dry corridor whereas Matagalpa is an upland area that is typically less severely affected, though still drought‐stressed. In Ethiopia, Kombolcha was categorised as in crisis/emergency well into 2016, whereas Seru was stressed/in crisis,^5^ and includes lowland areas that were affected by floods in the *kiremt* season.

Christian Aid has supported community‐based resilience projects in Nicaragua with local partners Centro Humboldt and Movimiento Comunal Nicaragüense/Matagalpa since 2011 (hereinafter referred to as ‘the projects'). This has built on earlier disaster risk reduction support through the application of participatory vulnerability and capacity assessment (PVCA),^6^ with interventions based on community risk management plans. A key feature has been access to climate services (weather and climate forecasts) to broaden anticipation to other climate risks, especially drought. Centro Humboldt has developed a downscaled regional climate model, supported by a community rain gauge network now expanded to over 100 measuring points. Climate forecast information is communicated to participating communities through a monthly Boletín de Monitoreo Climático, direct advisory support and SMS.

In Ethiopia, the areas assessed comprised two of seven included in the Climate Information and Assets for Resilience in Ethiopia (CIARE) project^7^ most severely affected by the drought, implemented by ActionAid Ethiopia. Unlike Nicaragua, they had benefited from only two years of resilience‐focused activities. This also includes building community resilience through the Building Resilience and Adaptation to Climate Extremes and Disasters (BRACED) Participatory Approach (BRAPA), which uses PVCA as its basic methodology but includes climate services from the outset based on assessments showing that climate risks typically make up 40–70% of the risk profile.

In both projects, a survey was deployed using KoboToolBox,^8^ asking a similar set of questions tailored to local context, cropping patterns and so on. In Nicaragua, a sample of 200 (80 project participants, PPs, and 20 non‐participating indirect beneficiaries as a comparison group (CG) in each area) was surveyed; in Ethiopia, 240 were selected – 80 participants from high‐intensity areas (HI) and 40 from medium‐intensity areas (MI) in each site – to enable an element of comparison between those involved in the project package of community‐based resilience‐building plus climate information services (high‐intensity) and those receiving only climate services (medium‐intensity), as well as between Kombolcha and Seru. A gender split of 60:40 women to men respondents applied to both countries.

The use of comparison groups was considered useful in improving the reliability of evidence despite the acknowledged challenges in the use of control groups for measuring resilience. In this study, it was clear that, in both areas, efforts were made to ensure drought information and support reached far beyond the direct project participants, as it should in such a situation. This made identification of a ‘control group’ impossible, whereas there were differences in intensity of support, albeit less precise, between direct/high‐intensity and indirect/medium‐intensity participants.

Twelve focus group discussions^9^ were carried out to enable interactive, qualitative discussion and triangulation with survey results. Six of these used timeline exercises as the basis for a structured discussion about the community's experience of the period before, during and after the drought. This enabled discussion of how conditions had unfolded and affected lives and livelihoods at each stage. The sequencing for both the survey and focus group discussions was designed around the risk cycle, to investigate first the alert phase to understand any early warning received and early action taken, then emergency relief and finally post‐drought recovery.

Both processes were complemented by key informant interviews, including with partner staff, the national meteorology agency and local government advisors. For quantitative measurement of crop yields, cost savings and damage avoided, the survey relied on participant/farmer post‐harvest estimates, which have demonstrated some reliability (Murphy et al., 1991).

## Livelihoods and access to resilience support

Livelihood profiles revealed a high dependency on agriculture across all of the sites, from 67% in Somotillo to 98.6% in Kombolcha. Livelihood diversity was higher in Nicaragua, with Matagalpa exhibiting the highest spread across eight options. Typically a quarter to a third of the profile was off‐farm compared with just 1.5–3.5% for Ethiopia. The lowest reliance on annual cropping was found in Kombolcha, largely related to the increase in the cultivation of *chat* as a drought‐resilient cash crop. Seru showed the lowest diversity, with 94% reliance on just two livelihoods: annual crops and livestock.

The main staples in western Nicaragua are maize and beans, with sorghum emerging as a drought‐resilient option. In Matagalpa, coffee is the main perennial crop, with some farmers switching to cocoa in response to longer‐term climate changes. Crop diversity is more marked between the two Ethiopian sites, with Kombolcha essentially following a maize/sorghum/*chat* system with some localised horticulture and Seru focusing on wheat/barley/maize/*teff*.

Access in Nicaragua to other resilience support by project participants either before or after the drought struck was limited. About three quarters had no other sources, with the exception of participants in Matagalpa, where nearly 30% accessed alternate sources of climate information. Around 70–80% received resilience support only from the project. Some assistance, covering up to 20% of respondents, was recorded from local government or other local NGOs with much lower levels recorded in Somotillo, where only other church agencies were significantly active. Any resilience gains were thus attributable largely to project activities. In Ethiopia, both local government advisory services and the Productive Safety Net Programme (PSNP)^10^ were more pervasive. In focus group discussions, respondents saw support from the projects and local government services as contiguous, which made clear attribution to anything other than a collective of these difficult.

## Access to early warning, forecasts and use of local information

Access to early warning of the drought varied across project sites and between countries. Most project participants in Nicaragua received their first early warning in April, whereas those in the comparison group were alerted two months later. By contrast, for participating households in Ethiopia, most of those in Kombolcha were notified in August whereas Seru received minimal warning. Only about a third of households in Kombolcha reported getting an early warning; the proportion dwindled to just 2% in Seru.

**Figure 2:**
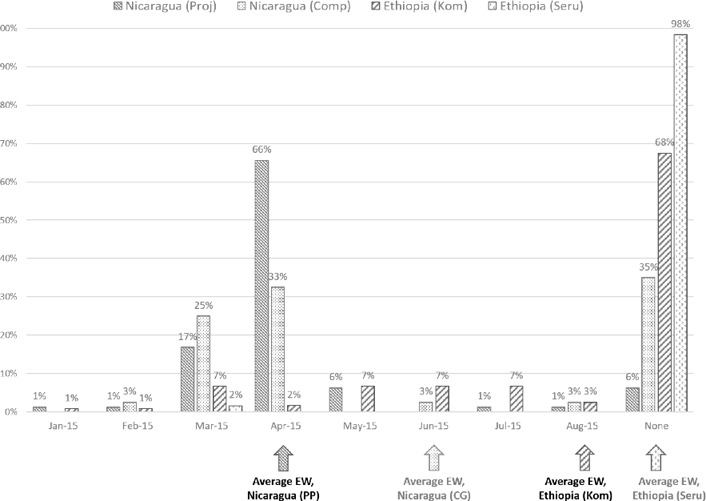
Timing of drought early warning **Source**: authors.

Sources of early warning varied across the different contexts. Project participants in Nicaragua relied largely on direct advice and the monthly newsletter, whereas the comparison group tended to use more generally available services such as radio and TV broadcasts. Community rain gauge data illustrated how some upland areas had a reasonable *primera* but a poor *postrera*, whereas the reverse applied in the dry corridor. Poor rainfall performance combined with higher than average temperatures, driven by El Niño and long‐term climate change, to amplify drought risks. In Kombolcha, about a quarter of households accessed local government agricultural advisors. In focus group discussions, respondents explained that they had relied on either local indicators such as wind direction or the failure of the *belg* season, signifying the onset of a dry year. While the survey showed only 2% access to early warning in Seru, the focus groups suggested better coverage of 24%, with 35% agreeing they had consulted a traditional weather forecaster or used local indicators. Discussions in Nicaragua confirmed the predominance of the project as a main source of early warning; in the case of Kombolcha and Seru, they confirmed the lack of early warning and only non‐specific drought forecasts received well after the onset of the *belg* and *kiremt* rains.

Use of local indicators or traditional forecasts was prevalent before the season started, particularly the behaviour of certain bird and tree species. Project participants in Nicaragua receiving more and more regular scientific forecasts were more likely to use local indicators before the season started – 74% versus only 50% of the comparison group. Focus groups explained the value of cross‐checking the forecast with local knowledge as a way of increasing user confidence. Use of local indicators during the season was significantly lower, at only 38%, suggesting a preoccupation with how and when rains arrive and overall seasonal performance. A similar pattern emerged in Ethiopia, with participants in Kombolcha reporting higher usage of local indicators and traditional forecasters, consulted by over 80% of respondents, than in Seru. Greater access to scientific forecasts in both Nicaragua and Kombolcha, combining with greater use of traditional forecasts and local indicators, suggests these are complementary rather than competing forms of decision‐making resources.

The picture with respect to how scientific forecasts were received before and during the season was mixed. In Nicaragua, while there was no difference with regard to one‐ to three‐day and seasonal forecasts between those in the project and those outside it, participants were much better served with weekly and monthly information, receiving 60–70% of this from project sources. This resulted in only 3% receiving no forecast information compared with 30% for the comparison group, although over 20% of this latter group still received monthly forecasts through the project. This suggests that the policy of ensuring as wide a coverage as possible beyond just direct participants was at least partly successful. About 98% of project participants received forecast guidance – a level that dropped only to 70% for the comparison group.

In Ethiopia, medium‐intensity areas in Kombolcha were better served with weekly and monthly forecast information, and more likely to have received these through the project, than high‐intensity areas. In Seru, the reverse applied, although high‐intensity areas in both locations were more likely to have received some seasonal forecast information. Overall, the only group receiving no seasonal forecast information was Seru medium‐intensity, with only 17% receiving any guidance at all, compared with 100% of high‐intensity participants receiving at least one forecast product.

The climate services component of the project was still under development in 2015 and has been deployed only since August 2016, so forecast provision in 2015 generally occurred through local government agricultural advisors or general radio forecasts. Given the low levels of forecast transmission to respondents in Seru through formal channels, it is possible that a significant part of the response refers to use of traditional or local knowledge. The relatively higher level of response here is also inconsistent with those for other forecast‐related questions. As no high‐intensity respondents were recorded in the ‘no forecast’ category, this could indicate some effects of the BRAPA process in sensitising households to their use.

Receiving early warning and forecasts did not necessarily translate into usefulness. In Nicaragua, project participants were more likely to rate both early warning and receiving forecasts before the season started as very useful or useful, with a majority of the comparison group agreeing. For Kombolcha, a high level of users found all sources of early warning and/or forecast information useful, whereas in Seru over half did not use formal information at all.

**Figure 3:**
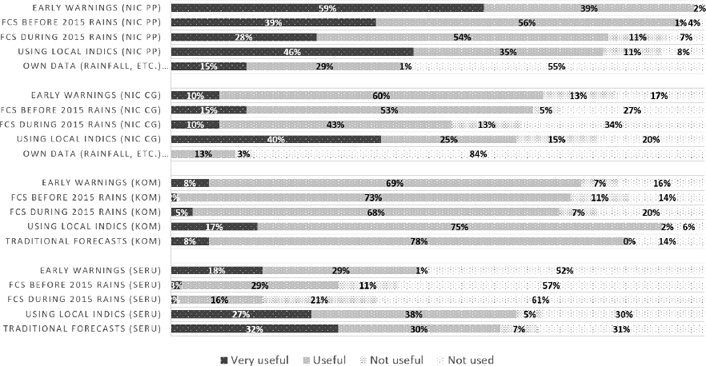
Usefulness of early warning and forecasts **Source**: authors.

## Access to and use of early action advice before and during the agricultural season

Advice on how to respond practically is generally seen as essential to effective drought resilience decision‐making. For project participants in Nicaragua, this included guidance on planting timing and choice of crops and crop varieties, which reached 60–75% of producers. A second category in the 20–40% range of producers included land preparation, use of fertilisers, crop pests to expect and whether or not to plant in the *primera*, a message aimed primarily at farmers in the dry corridor. Recipients of this advice confirmed decision‐making in the same proportions, although 44% also reduced the area cultivated while a small number increased it.

Changing crop or crop variety was favoured over increasing the area for drought‐resilient cropping, suggesting that farmers used strategies to increase drought‐resilient crops together with limiting cultivated area to concentrate resources. Changing planting date was the most popular focus, but the ‘after season start’ results showed that this could be earlier or later. Focus groups explained how rain gauges and soil examination were used to determine when to trigger planting – for example, 60 mm falls within three days, with soil soaked to a 10 cm depth.

Decision changes made during the season tended to be less pronounced, with only three – planting later than usual, changing pest control methods and applying more fertiliser – being changed by more than 30% of farmers. For both before and in‐season decision‐making, the difference between project participants and the comparison group was more pronounced than for receipt of early warning and forecast information, with 96% of the former changing decisions before the season started and a further 86% during the season, as against over 50% of the comparison group making no change as compared with a normal season.

In general, increased access to early warning, forecasts and drought resilience advice facilitated decision‐making, with nearly 70% of project participants making earlier decisions than normal. However, a significant minority delayed decisions until significantly later in the season. Project participants were more likely to make decisions significantly earlier and significantly later than the comparison group. Focus groups suggested later decision‐making was not necessarily a failure of the process but rather a conscious action by those in or near the dry corridor to suspend activities in the *primera* in response to forecast information from both official government and project sources, avoiding the likely losses and focusing resources on the *postrera*. In the event, this proved successful as, according to the groups, 90–95% of dry corridor farmers saved inputs – seed was also highlighted as especially difficult to find during the season – and costs they would have otherwise incurred.

About 45% of comparison group farmers also stated that the timing of their decisions was significantly or slightly earlier, but this was more likely the result of some access to early warning and forecast information rather than because of resilience‐building advice. The relatively low numbers in the comparison group highlighting the importance of resilience advice to the use of early warning/forecasts reflect their lower access to this information. The reverse was evident among project participants, with 86% declaring it either essential to or greatly improving their ability to use early warning and forecasts effectively. Focus groups confirmed this but added more detail: the use of diversification across both field and horticultural crops to strengthen drought resilience; intensifying production and water conservation techniques on smaller plots; and sourcing a drought‐resilient bean variety – *frijoles alacin* – to address increasing concerns that red beans (*Phaseolus vulgaris*) had proved vulnerable to increasingly erratic and drought‐prone growing conditions.

For project participants in Ethiopia, access to drought‐resilient agricultural information was broadly similar for both high‐ and medium‐intensity areas but varied considerably between Kombolcha and Seru. In Kombolcha, 75% of farmers received some advice on use of crop varieties and 60% confirmed guidance on which crops and when to plant. Just under 40% were advised on harvest timing and 30% on land preparation techniques. Smaller numbers, in the 10–15% range, reported guidance on soil fertility, pests and water resource management. For decisions changed, altering planting time was the most popular strategy, with over 40% of farmers planting earlier but a significant 20% also planting later, suggesting that, as with the Nicaraguan respondents, advice had generated some flexibility. The second most important change category covered choice of crops, with 31% changing crop variety but a lower 18% changing crop.

A reduction in planted area was confirmed by only 15%. Focus group discussions in Kombolcha placed more emphasis on this tactic, with respondents highlighting that they planted only in areas used for dry season cultivation, such as river banks. Changes generally conformed to the advice received but only 2% of those surveyed indicated that they had prepared their land differently, despite 27% confirming advice on how to prepare their land. This suggests this advice was not sufficiently actionable.

Decisions changed after the season had started emphasised earlier harvesting, which confirms the popularity of earlier planting but also changing to early‐maturing crop varieties. This is something that focus groups stressed, although they also expressed some reluctance to adopt early‐maturing sorghum, as they value their traditional long‐stem varieties as much for livestock feed as for grain production. Interestingly, only 6% explicitly confirmed increasing the area of drought‐resilient crops. Increased and changing pest control methods occupied 25–30% of information users, while over 25% used irrigation more intensively. Coupled with reducing area cultivated, this represents an important coping strategy. Although only small numbers registered change, it was more common to reduce fertiliser application than to increase it.

For Seru, more than 10% of respondents received no category of advice, and, for decisions changed before or during the rainy seasons, more than 5% of farmers cited only reduced cultivated area and changing crop. Focus groups in Seru added some detail, suggesting that drought mitigation was on the agenda both before and during the *belg* and *kiremt*. Three out of four groups dug or expanded rainwater catchment ponds, and all emphasised a switch to drought‐tolerant crop varieties through seed supplied by the project, especially for the *kiremt* season. However, this translated into a much higher level of unchanged decision‐making than was the case for Kombolcha.

### Impact of early action on productivity, input use efficiency and damage avoided

Assessment of productivity changes sought to understand whether project participants had achieved yield improvements as compared with a normal year, with a previous drought year and with those benefiting indirectly (as with the comparison group in Nicaragua) or from only a part of the full project implementation (medium‐intensity households in Ethiopia). For Nicaragua, maize, beans and sorghum were assessed in both assessment sites; in Ethiopia, cropping patterns varied, with maize, sorghum and *chat* assessed in Kombolcha and wheat, maize and *teff* in Seru.

Results reveal a diversity of performance. In Nicaragua, project participants reported a substantial improvement for maize as compared with the previous 2014 drought. Maize yields were almost at levels expected for a normal year and 72% higher than those for the comparison group.^11^ The difference between 2014 and 2015 could partly reflect slightly more favourable growing conditions in some locations as focus groups (especially in the drought corridor) reported unusually late rains in the *postrera* that rescued what would otherwise have been a much worse season. However, the difference between project participants and the comparison group suggests that about two thirds of the yield resilience could have derived from early warning, early action and drought‐resilient decisions (without considering the indirect support the comparison group received). Yield difference for beans was less distinct and no difference was recorded with the comparison group for sorghum. Many farmers were growing sorghum for the first time and only about half the comparison group grew it, making it difficult to assess any differences reliably. Beans are known for their increased vulnerability to drought, so it is possible that resilience measures would have had less impact.

**Table 1:** Yield comparisons

Crop	(a) PP/HI 2015 % diff vs. normal year	(b) PP/HI 2015 % diff vs. previous drought year	(c) PP/HI 2015 % diff vs. CG/MI 2015
**Nicaragua**			
Maize	−1.3	**+238.4**	**+71.6**
Beans	−44.7	**+62.5**	**+6.6**
Sorghum	−38.4	**+73.8**	**+0.5**
**Ethiopia (Kombolcha)**			
Maize	−37.5	**+546.8**	**+44.8**
Sorghum	−43.3	**+869.3**	**+46.3**
Chat	−49.5	**+311.5**	−230.8
**Ethiopia (Seru)**			
Maize	−79.7	−38.4	−4.9
Wheat	−81.8	−34.5	−53.9
Teff	−77.2	−12.8	**+2.3**

**Source**: survey results.

For Kombolcha, there was some difference in maize yields comparing high‐ and medium‐intensity areas. Although nearly 40% lower than a normal year, they were still 45% higher in high‐intensity areas and very substantially higher than in the previous drought 10 years earlier.^12^ Sorghum showed the same pattern as maize, with an over 40% decline on normal yields but a 46% increase for high‐intensity areas. As access to early warning and forecast information did not vary between high‐ and medium‐intensity areas, and drought resilience advice was similarly evenly spread, with better access for medium‐intensity households in some cases, the better performance of high‐intensity farmers for maize and sorghum may result from other factors, such as better preparedness through BRAPA and resilience action plans, less focus on cash crops such as *chat* and higher levels of access to the PSNP.^13^ In Seru, low levels of early warning and drought resilience advice translated into very little difference between high‐ and medium‐intensity areas and yields lower than were experienced in 2005/06.

Comparing all areas assessed on maize production demonstrates the combined value of early warning, good access to forecasts and applicable drought resilience advice, with almost average yields for six years of resilience‐building in Nicaragua and some improvement based on ongoing advisory support and initial project activities in Kombolcha but no real change in Seru. Although maize shows this most clearly, results for sorghum also demonstrate some impact. Focus groups tended to emphasise productivity impacts extending to other crops, such as beans, and through diversification into vegetable production, in both countries. Yield improvements should be considered together with area cultivated, given that over 40% of project participants in Nicaragua and at least 15% in Kombolcha reduced the area they cultivated, enabling the concentration of resources. This would clearly reduce overall productivity.

**Figure 4:**
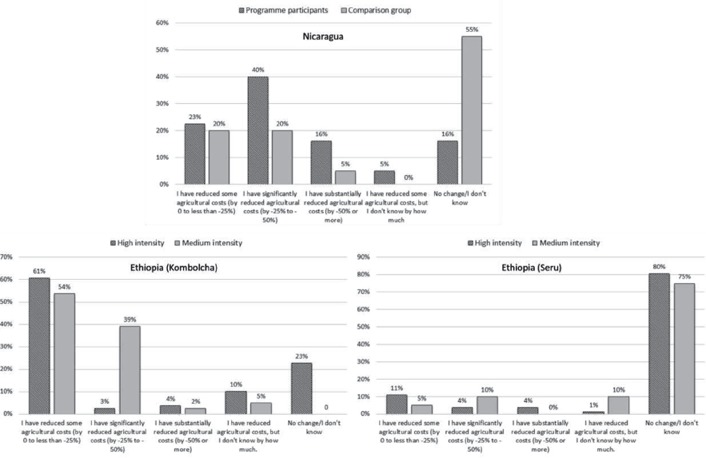
Input cost changes **Source**: authors.

In Nicaragua, these benefits also reflected input use efficiency, with 84% of respondents indicating some level of input cost reduction – roughly twice the number reducing cultivated area, suggesting forecasts and resilience‐building advice had contributed to more effective input use. Focus group discussions confirmed this, pointing to measures such as better timing of crop planting using forecast and rain gauge information and more efficient use of water resources, manures and crop residues. Interestingly, more or less the same proportion of project participants and comparison group farmers felt they had reduced some costs (0–25%) but the difference widened for both significant and substantial cost reductions. This may also reflect the value added of effective early warning/early action over and above a reduction from limiting cultivated area.

Results suggest that, in Kombolcha, respondents reduced input costs through more than simply reducing agricultural area, but there was little difference between high‐ and medium‐intensity areas: 78% and 100% of respondents confirmed savings and medium‐intensity areas were more likely to be in the higher ‘significantly reduced, ‐25% to ‐50%’ category. Conversely, in Seru, around 80% of farmers in both intensity categories saw no change in costs.

Damage avoided is the third category of direct impact and typically the hardest for project participants to assess, as it is an assessment of something not happening. In Nicaragua, while 60% of the comparison group saw no reduction, 48% of project participants felt they had managed to mitigate substantial damage to crops and livestock, with a further 30% able to achieve significant or some reduction. In Kombolcha, over 60% of high‐intensity participants felt they had avoided some damage but over 80% in medium‐intensity areas registered significant or substantial damage reductions whereas over 70% in Seru saw no change. These results were consistent with other areas of impact.

## Access to emergency relief and recovery

Although no emergency was declared in Nicaragua, some areas received drought relief, which, according to the focus group discussions, tended to be targeted to vulnerable households or children after formal assessments. It was difficult to discern a systematic pattern – the number of distributions varied from village to village, but groups in the dry corridor particularly emphasised the importance of PVCA‐based action plans, submitting these to municipalities to request and access drought relief. This was verified by survey results, with action plans said to have strengthened drought response across a variety of criteria, especially who to contact in the event of drought, what to do after an early warning, who is most vulnerable and how to respond. In the event, participants had better access to drought relief but mainly because of their relationship with the project, which provided the majority of support. Around 80% agreed that the action planning process had improved understanding of what relief actions to take in response to both early warning and forecasts of El Niño and drought, as well as highlighting more vulnerable members of their community.

The emergency response in Ethiopia was substantially larger, with over 90% of respondents in both high‐ and medium‐intensity areas in Seru and nearly 60% of high‐intensity respondents in Kombolcha receiving drought relief. However, according to focus groups, the main distribution started in February 2016, some eight months after the first *belg* harvest failure of the drought. Earlier food distributions occurred, so in Seru one group highlighted PSNP‐related food distributions starting in August and continuing through to 2016, together with NGO‐supplied animal feeds and water in June. However, three of the four groups highlighted the need to migrate in search of food and livestock‐grazing, or to look for casual work to earn enough to buy food, suggesting a need for earlier/more substantial drought relief.

In both areas, discussions emphasised the usefulness of the BRAPA action plans in drought response, including protecting water sources, increasing access to irrigation, focusing on vegetable‐growing and accessing emergency loans through savings and credit groups to sell, for example, livestock and eggs to earn money to buy food. These issues were also apparent in the survey results. Although less emphatically than in the responses in Nicaragua, participants in Kombolcha indicated slightly better levels of registration of action plans with local government and other agencies. About 95% agreed with the statement that their action plan identified what to do in the event of drought. For Seru, the picture was more evenly balanced, with agreement responses generally about double the score (around 40%) for those disagreeing, but 35–40% in the ‘don't know’ category. Responses related to knowing what to do in the event of a drought, registration of the plan with local government and NGOs and identification of the most vulnerable registered slightly higher scores.

It was not the aim of this study to explicitly understand the role of safety net programmes in drought resilience, and these are present in only one of the two countries, but the PSNP does make up a significant part of the context in Ethiopia. A number of reports highlight the need to expand social safety nets as a way of enhancing the resilience of the poorest.^14^ In this assessment, it seems to have been most clearly cited by communities as a drought relief mechanism covering the period between emergency onset and the arrival of the main emergency drought relief programme, rather than as a source of early warning and drought risk mitigating support. Given the eight months that it took from the failure of the 2015 *belg* harvest to the delivery of the main drought relief programme, this earlier drought relief was clearly an important safety net function. It may also have played a role in enabling high‐intensity area participants to achieve slightly higher yields than medium‐intensity areas, although responses linked this more clearly to local government extension advice and project support.

Key informant interviews also revealed the difficulty in providing early warning or early action advice in the face of oncoming drought, suggesting that this could not happen in the absence of an official declaration of emergency. To act before this would have negative repercussions for the early warning provider. In comparison, much higher levels of early warning and early action delivered greater resilience and resulted in only 30% of project participants accessing drought relief in Nicaragua, with no safety net support available.

**Figure 5:**
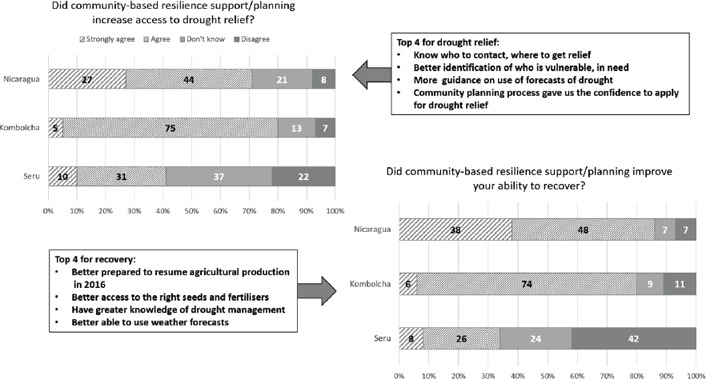
Access to drought relief and post‐drought recovery **Source**: authors.

The difference in assessment of recovery between the three areas is consistent with earlier results on access to forecast information, yields, reduced input costs and damage avoided. Between 30% and 60% of participants in Nicaragua strongly agreed that their capacity to recover had been enhanced by resilience‐building support across eight recovery factors, with particularly strong responses for being able to resume agricultural production and better access to the right seeds and fertiliser. Conversely, food security and financial security recovery received lower scores than other measures, a pattern also evident in the results from Kombolcha. This reflects the unavoidable loss and damage that droughts can cause and the need for supplementary support, such as appropriate interventions to enhance early recovery processes. These might entail maintaining increased safety net‐based transfers and increasing access to community‐based savings and loans systems. The limited results of resilience‐building so far in Seru were reflected in a much less optimistic assessment of recovery, with only 35–40% of participants indicating improved circumstances versus over 40% considering themselves worse off.

## Discussion

The results demonstrate the value of both long‐term resilience‐building through community‐based risk assessment and action planning processes and timely access to drought early warning and early action advice in mitigating drought risk associated with El Niño in both countries. In particular, the difference between households facing drought in Nicaragua after five to six years of resilience‐building and those in Ethiopia with only one to two years suggests that providing early warning alone is necessary but not sufficient. The early warning/early action dividend depends on the development of a resilience culture through long‐term strengthening of resilience planning and forecast use capacity.

Community involvement in collecting data through rain gauges and providing this information resource for the development of forecasts and drought resilience advice in Nicaragua contributed to its effectiveness through increased understanding of forecasts, ownership of the forecast development process and motivation to apply early warning and forecast information. Local data collection also adds to the drought resilience tools that communities can apply autonomously, such as the use of rain gauge data to guide planting times. This results chain of long‐term resilience‐building leading to more effective, lower‐cost early warning and early action, which then leads to more effective, lower‐cost humanitarian response can be summarised as follows.

In terms of overall productivity, yield results need to be considered together with reducing area cultivated as a drought response strategy. While focus group discussion outcomes in Nicaragua triangulate well with the survey results in terms of access to early warning, forecasts, resilience advice and crop yields, a number of differences emerged in Ethiopia. In Seru, discussions suggested better, although still relatively low, access to early warning and use of forecast information than did survey results. In Kombolcha, focus groups tended to describe the drought‐affected seasons as failed harvests, whereas survey responses indicated some, albeit lower than normal year, productivity. Comparing yield differentials across three measures suggests that the combination of timely early warning, forecasts and drought resilience advice does result in yield reductions compared with normal years but better performance compared with previous drought years for some main crops (maize, sorghum) and those receiving direct support coping better than indirect participants.

Input cost reduction in Nicaragua reflected a similar pattern, with 84% of participants indicating some level of decrease – roughly twice the number indicating that they had reduced cultivated area and twice the number recorded in the comparison group. This suggests a net benefit over and above simply reducing cultivated area as a response, something also confirmed by focus groups, which cited reduced seed costs, better use of crop residues and avoidance of agrochemical inputs as resilience‐building measures. Kombolcha showed a similar pattern with a lower but still positive response, and Seru no real change. Measures of damage avoided followed a similar pattern.

Yield increases for maize of over 70% in Nicaragua and 45% in Ethiopia (Kombolcha) compare with a typical 10–20% enhancement through increased access to forecasts in normal years (see Christian Aid, 2015). This suggests that, while increased access to forecast services is effective in normal years, their importance increases in severe drought years.

In the case of Nicaragua, an emergency was not declared and emergency relief was highly targeted; in Ethiopia, the emergency declaration process is well established but this assessment found only very limited access to early warning and drought forecasting beforehand. The main programme of emergency relief started in early 2016, several months after the actual drought emergency in communities had begun (with the 2015 *belg* season failure). This resulted in significant hardship and asset loss that could have been mitigated. Effective early action by civil society organisations delivered drought resilience that communities highly valued. This could have been substantially enhanced in both areas assessed if public sector agencies had had similar systems that enabled cooperation and productive, coordinated implementation of early warning/early action‐based drought resilience programming before the emergency started.

While part of the value of the assessment has lain in comparing an intervention in Nicaragua receiving long‐term resilience support with one in Ethiopia at an earlier stage of this process and a track record of more conventional development and humanitarian assistance, comparison needs to take into account wealth, developmental and risk exposure disparities. In Nicaragua, gross national income per capita is $1,940; in Ethiopia it is only $590;^15^ Nicaragua is 124^th^ on the Human Development Index; Ethiopia places 174^th^. On the other hand, Nicaragua ranks 24^th^ on the Climate Risk Index (Kreft et al., 2017) for 2015 whereas Ethiopia is 65^th^; considering the wider 1996–2015 period, Nicaragua ranks 4^th^ compared with Ethiopia's 66^th^. More initial livelihood diversity in Nicaragua could also translate into higher resilience. Overall, these differences could result in over‐estimating the difference in the results of resilience‐building between Nicaragua and Ethiopia.

## Conclusion

Drought‐affected communities made a wide variety of decision changes in response to early warning, forecast and drought early action advice. These related to planting time, choice of drought‐resilient crops and crop varieties, changing area cultivated and, in Nicaragua, not planting with the first rainy season. This was important in managing drought risk. The impact achieved in both Nicaragua and Ethiopia is consistent with that found by other studies on the cost/benefit of resilience. With both climate change and the cost of humanitarian intervention increasing annually, these results demonstrate the need to transform the current approach from emergency declaration/late response to early warning/early action.

Soil and water resource management is a key drought resilience measure but receives lower levels of attention: only 30% of participants in Nicaragua and 2% in Ethiopia (Kombolcha) prepared land differently before the onset of rains. This suggests scope for greater focus that will also yield dividends in normal years. Focus group discussions in all areas referred to the need for, for example, community ponds, more resilient wells and better soil moisture management. This suggests they are also considered a priority for local action. Other options, including conservation agriculture, agroforestry and terracing, would ensure vulnerable farmers could benefit further.

Early action advice in bimodal systems with rainy seasons beginning in the first half of the year needs to manage the uncertainty in the forecasting system. Ensuring early warning and forecast advice for both growing seasons is effective means the earliest possible warning advice for the *primera/belg* and the *postrera/kiremt* growing seasons. A potential drawback is the increased uncertainty of ENSO forecasting in the first four months of the year, before the ‘spring barrier'. This means forecasts for the *primera/belg* that already have a shorter lead time are also less reliable. Forecast users need to be aware of these issues so they can calibrate drought resilience decisions according to the levels of regrets they may contain.

Advice that has potentially significant high regrets needs to be managed carefully. For example, in Nicaragua, most farmers in the dry corridor received advice that suggested not planting in the *primera* but concentrating resources on the *postrera*. In the event, this proved good advice and saved those who followed it from significant input losses. Farmers’ own rainfall data and the use of the rainfall/soil moisture threshold to guide planting time also assisted in this decision. However, if rains had arrived, lost production could have been considerable and reduced user confidence in early warning/forecast information, especially if the subsequent *postrera* had failed completely. This highlights the need, as the implementing agencies achieved through the use of PVCA/BRAPA, to emphasise the sovereignty of farmer decision‐making, ensuring they are aware of forecast skill, uncertainty and potential risks in using probabilistic forecasts, maintaining a continuous flow of information so actions can change as conditions evolve. Drought resilience‐building processes are strengthened when communities receiving advice are supported to assess and understand the management implications and the degree to which low‐ or high‐regret actions are possible. This enhances their analytical capacity, their management of forecast uncertainty and their flexibility in tailoring decisions to their own enterprises, recognising that each has different asset endowments and family and social circumstances.

**Figure 6:**
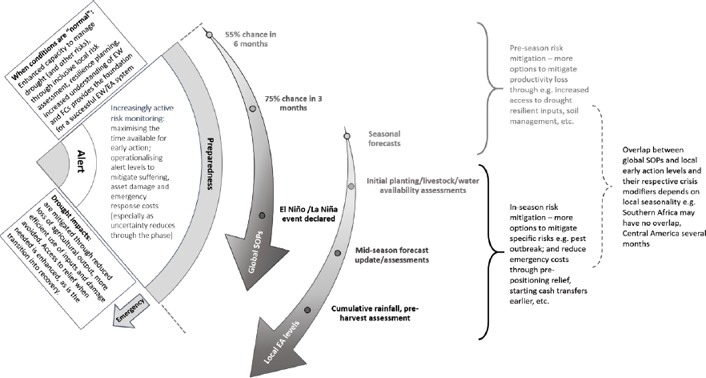
Long‐term resilience‐building enhances early warning/early action, which increases the efficiency of humanitarian response **Source**: authors.

Responses reflecting on the usefulness of community‐based resilience planning suggested that this process enhanced access to drought relief and, together with the improved resilience gained from early warning and early action, had enabled project participants to recover more successfully after the drought ended. Aggregating the recovery factors investigated (in figure 6 above) showed a progression from very positive in Nicaragua to neutral in Seru, reflecting and consistent with the other impacts detected. Interestingly, Nicaragua and Kombolcha showed a similar pattern of response, with both food and financial security lagging behind other more positive recovery characteristics.

While the BRAPA planning and resilience support had already delivered some positive outcomes in Kombolcha, six years of resilience‐building in Nicaragua demonstrates what can be achieved through more persistent support for early warning, early action and post‐drought recovery. That in both countries, whatever improvements to the efficiency of emergency response have been delivered, there remains no formal early warning/early action system and process in operation suggests that the situation with respect to mitigating drought risk remains underdeveloped.

These results show the need for climate funds at global and national levels to focus on delivering resilience through localised, community‐led climate risk planning and resilience‐building actions. Drought resilience for the most vulnerable needs long‐term and consistent support to be built and maintained by all stakeholders, including climate service providers, local government and civil society. As climate change intensifies future droughts, the importance of this community‐based anticipatory approach will grow. More information is needed with respect to the long‐term implications of climate change on the ENSO cycle and the consequences for both humanitarian and early warning/early action procedures and processes.^16^ The relatively low levels of project participants acknowledging that they had received or understood long‐term climate change scenarios in Ethiopia suggests increased use could be beneficial.

Long‐term support needs to be complemented by a clear forecast‐based system of early warning and early action levels. Taking the alert stage from the risk cycle shows how ENSO forecasts, here using the United Nations Food and Agriculture Organization/Office for the Coordination of Humanitarian Affairs global early warning levels (IASC, 2018), can be combined with more locally specific information resources to define early action thresholds. Once the season starts, forecast‐based early warning is increasingly supplemented by ongoing assessment of the actual situation. As these progress, uncertainty is reduced and the level of low‐regret drought mitigation action can be increased at each stage. It is the successful operationalisation of this type of system, based on a long‐term foundation of increased resilience and capacity, that successfully mitigates drought risk.^17^

**Figure 7:**
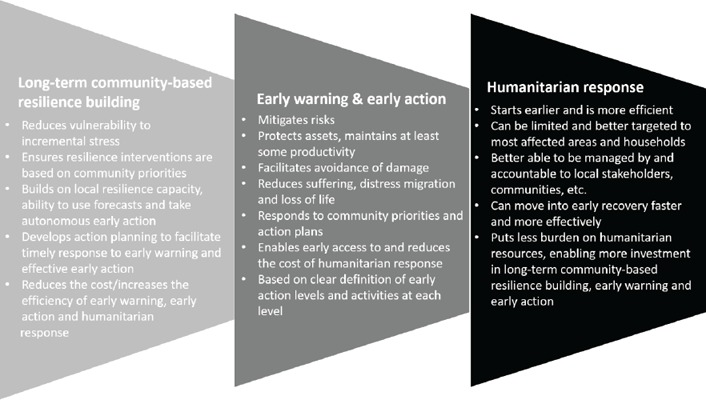
Combining global El Niño‐based early warning with local early action thresholds in the alert stage **Source**: authors.

This reinforces earlier conclusions with respect to the need for a more integrated approach to addressing short‐term shocks, long‐term stresses and resilience: ‘*the separation of relief and development is both artificial and unhelpful. Not only are the recipients the same, but also the underlying causes that create the need are the same*’ (Cabot Venton et al., 2012).

## Acknowledgements

The authors would like to thank the managers and staff of Christian Aid Central America, Centro Humboldt, Movimiento Comunal Nicaragüense, Christian Aid Ethiopia, BBC Media Action and the National Meteorology Agency of Ethiopia for their support in carrying out this study; and the 440 farmers, 12 focus groups and 13 enumerators who related and collected experiences of the 2015–16 El Niño‐related drought.

## Endnotes

2 The Global Fund has received $48 billion to date (see https://www.theglobalfund.org/en/portfolio/); the GEF has received nearly $21 billion over six replenishment cycles since 1994 (see https://www.thegef.org/about/funding); the CERF has received $5.3 billion in contributions since 2006 (see http://www.unocha.org/cerf/donors/donorspage).

3 With a peak strength of +2.6 in December, comparable to the very strong El Niño of 1982–83 (+2.2) and that of 1997–98 (+2.4), the 2014–2016 event was the longest‐lasting and strongest on record.

4 ENSO forecasts – synopses from ENSO Diagnostic Discussions issued monthly by the Climate Prediction Center and the International Research Institute for Climate and Society (February 2015–August 2016). Rainfall data from Africa Flood and Drought Monitor and Centro Humboldt community rain gauge records across Nicaragua.

5 As per Integrated Phase Classification (IPC) levels: 2 = stressed; 3 = crisis; 4 = emergency; 5 = famine. See http://fews.net/IPC

6 PVCA is carried out in a community to collect, analyse and systematise information about its vulnerability and capacity in a structured way. Its main purpose is to identify the key vulnerabilities of a particular community; understand how community members perceive risks (shocks and stresses) to their lives and livelihoods; analyse the resources (capacities) and strategies available to them to address or reduce these risks; and help the community develop an action plan they will implement with support from other stakeholders (Christian Aid, 2009).

7 CIARE is a project under BRACED, or Building Resilience and Adaptation to Climate Extremes and Disasters, a UK Department for International Development (DFID)‐funded programme working through 15 projects across Africa and Asia. See http://www.braced.org/

8 See http://www.kobotoolbox.org/

9 Six per country, three per site. Although focus groups in Kombolcha were facilitated by the assessment team, the State of Emergency in Ethiopia meant time limitations prevented access to Seru. ActionAid Ethiopia staff carried out the process there.

10 Established in 2005, the PSNP aims to enable the rural poor facing chronic food insecurity to resist shocks, create assets and become food self‐sufficient. It provides multi‐annual predictable transfers, as food, cash or a combination of both, to help chronically food‐insecure people survive food‐deficit periods and avoid depleting their productive assets while attempting to meet their basic food requirements. See https://www.wfp.org/sites/default/files/PSNP%20Factsheet.pdf

11 Average estimated maize yields for a normal year are close to the average yield for Central America (2009–13: 2,206 kg/ha) but higher than that recorded for Nicaragua (530 kg/ha) (World Bank and CIAT, 2015).

12 Care needs to be taken here given the difficulty of recalling performance in a drought year a decade ago.

13 High‐intensity areas were chosen using data showing their relatively higher poverty and vulnerability, which also increases the likelihood that they are registered with the PSNP.

14 For example, Slater and Ulrichs (2017) conclude that, ‘at a systems level, anticipatory capacity is high'.

15 See http://data.worldbank.org/country/

16 While the frequency of strong El Niño events has been relatively constant at about one per decade, they have increased in strength for each decade since the 1950s (Climate Prediction Center, 2018).

17 See Christian Aid (2017) for the infographic research summary.
